# l-arginine supplementation reduces cardiac noradrenergic neurotransmission in spontaneously hypertensive rats

**DOI:** 10.1016/j.yjmcc.2009.03.023

**Published:** 2009-07

**Authors:** Chee-Wan Lee, Dan Li, Keith M. Channon, David J. Paterson

**Affiliations:** a Department of Physiology, Anatomy and Genetics, University of Oxford, UK; b Department of Cardiovascular Medicine, University of Oxford, UK

**Keywords:** Spontaneously hypertensive rat, l-arginine, Norepinephrine, Nitric oxide, Tyrosine hydroxylase

## Abstract

Spontaneously hypertensive rats (SHR) are known to have cardiac noradrenergic hyperactivity due to an impaired nitric oxide (NO)–cGMP pathway. We hypothesized that dietary l-arginine supplementation may correct this autonomic phenotype. Male SHR and Wistar Kyoto rats (WKY) aged 16–18 weeks were given l-arginine (10 g/L in drinking water) for 1 week. Separate control groups received no supplementation. The SHR control had a significantly lower plasma l-arginine than WKY control, but this was increased to a comparable level following l-arginine. Atrial cGMP was lower in the SHR control compared with the WKY control (2.4 ± 0.4 pmol/mg vs 3.9 ± 0.5 pmol/mg, *p* < 0.05), but increased to 4.1 ± 0.5 pmol/mg protein (*n* = 8, *p* < 0.05) with l-arginine. Evoked [^3^H]norepinephrine release in isolated spontaneously beating right atria from the SHR control (328 ± 19%, *n* = 19) was 28% higher than the WKY control (256 ± 20%, *n* = 14, *p* < 0.05), but was reduced to 258 ± 11% with l-arginine feeding (*n* = 24, *p* < 0.01). Soluble guanylyl cyclase (sGC) inhibition caused a greater increase of evoked norepinephrine release in the l-arginine fed SHR compared with the non-fed SHR. l-arginine feeding did not reduce evoked norepinephrine release in the WKY. In-vitro heart rate response to exogenous norepinephrine (0.1–5 μmol/L) was similar between l-arginine fed (*n* = 13) and non-fed SHR (*n* = 10), suggesting that l-arginine supplementation worked pre-synaptically. Myocardial tyrosine hydroxylase protein was decreased in SHR following l-arginine supplementation, providing a link to reduced synthesis of norepinephrine. In conclusion, l-arginine supplementation corrects local cardiac noradrenergic hyperactivity in the SHR, probably via increased pre-synaptic substrate availability of NOS–sGC–cGMP pathway and reduced tyrosine hydroxylase levels.

## 1 Introduction

Impairment of cardiac autonomic function characterises many cardiovascular disorders. Its presence is associated with adverse clinical outcome, even in apparently normal subjects [1,2]. It is well established that hypertension is strongly associated with noradrenergic hyperactivity, with increased central sympathetic output as well as elevated plasma epinephrine and norepinephrine (NE)^1^ levels [3–5]. Although the exact causal role of the sympathetic activation remains to be clarified, disruption of nitric oxide (NO)–cyclic guonosine monophosphate (cGMP) pathway modulating the sympathetic function either centrally or peripherally has been implicated [6–8]. We recently demonstrated that a significant component of cardiac sympathetic hyperactivity in spontaneously hypertensive rats (SHR) occurs at the end organ level with impaired NO–cGMP signalling, and that gene transfer with a noradrenergic cell specific promoter coupled to neuronal nitric oxide synthase (nNOS) could increase the production of cGMP with resultant inhibition of sympathetic neurotransmission in atria [9]. However, gene transfer involving adenoviruses is an invasive procedure and poses considerable hurdles to widespread use in clinical practice. We therefore explored a simple non-invasive method of augmenting the NO–cGMP signalling and examined its effect on local cardiac noradrenergic neurotransmission in the SHR in order to provide further basis for future clinical trials.

l-arginine (l-arg) is the sole substrate of the enzyme NOS in the synthesis of NO [10,11]. Intracellular concentrations of l-arg normally greatly exceed the level that is required for maximal enzyme kinetics of NOS; the Michaelis constant (Km) of l-arg for NOS is 2.9 μmol/L whereas the intracellular level of l-arg in-vivo is 0.8–2 mmol/L, with plasma concentration in the region of 60–350 μmol/L [12,13]. Despite this, l-arg supplementation has been shown to improve endothelial function, reduce atherosclerosis progression, and alter autonomic function; all consistent with enhanced NO synthesis [12,14,15]. This is termed the l-arginine paradox, and can probably be best explained by compartmentalisation of NOS and a relative l-arg deficiency rather than an absolute one.

In the present study, we showed that oral l-arg supplementation could reduce local cardiac noradrenergic hyperactivity in the SHR by increasing pre-synaptic substrate availability of the NOS–sGC–cGMP pathway, and provided further mechanistic link with demonstration of reduction in cardiac tyrosine hydroxylase levels.

## 2 Materials and methods

### 2.1 Animals

Male SHR aged 16–18 weeks and Wistar Kyoto rats (WKY) were sourced from Harlan, UK, and kept under standard laboratory conditions. l-arg supplemented groups were given l-arg (Sigma, UK) in drinking water (10 g/L water) for 1 week. Control groups received no supplementation but otherwise have free access to water and rat chow. The experiments conformed to the Animals (Scientific Procedures) Act 1986 (UK) and the Guide for the Care and Use of Laboratory Animals published by the US National Institutes of Health (NIH Publication No. 85-23, revised 1996).

### 2.2 Heart rate and blood pressure measurement

The heart rate and arterial blood pressure were measured invasively as a terminal procedure (prior to in-vitro physiological experiments) on some rats. Briefly the rat was ventilated under anaesthesia (3% isoflurane and 100% oxygen) and its left carotid artery was cannulated with a 3F portex cannula. The cannula was then connected to a pressure transducer and data was acquired using a Biopac M100 system connected to a Dell P4 computer and run on the AcqKnowledge 3.7.3 software. After an equilibration period of at least 10 min at lower isoflurane concentration (1.5%), the heart rate and blood pressure readings were taken. Following completion of the hemodynamic measurement, the animals were euthanised with intraperitoneal injection of pentobarbitone. Blood samples were taken via intraventricular puncture and the whole heart removed. Blood samples were immediately spun at 5000 rpm for 10 min. Red blood cell and plasma (in heparinised tube) were separated and stored, as was serum. These were snapped frozen in liquid nitrogen before being stored at − 80 °C for long term.

### 2.3 Local norepinephrine release from right atria

The spontaneously beating right atrium (RA) was isolated and transferred to a preheated (37 ± 0.2 °C), continuously oxygenated (carbogen: 95% oxygen, 5% CO_2_), water-jacketed organ bath containing 3 ml Tyrode solution (NaCl 120, KCl 4.7, MgSO_4_ 1.2, KH_2_PO_4_ 1.2, NaHCO_3_ 25, CaCl_2_ 2, and glucose 11 mmol/L, pH 7.4) where the atrium was pinned flat on a silver stimulating electrode. The method for determining the local NE release was based on one previously described with modification [9]. Briefly after a 20 minute equilibration period (during which the Tyrode solution was replaced every 10 min), the preparation was incubated for 30 min with 5 μl [^3^H] NE (0.185 MBq, Amersham) and ascorbic acid (30 μmol/L, Sigma). To facilitate the uptake and exchange of [^3^H] NE into the transmitter store in pre-synaptic terminal, the atrium was stimulated at 5 Hz (15 V, 1 ms pulse width) for 10 s every 30 s, for 30 min. After that, excess radioactive NE was washed from the preparation by superfusion with Tyrode solution for 45 min at a rate of 3 ml/min. Superfusion was then stopped and the bath solution was replaced every 3 min. 0.5 ml sample from each solution change was added to 4.5 ml scintillation liquid (Ecoscint A, National Diagnostics) and the amount of radioactivity (disintegration per minute, DPM) was measured using a liquid scintillation counter (Tri-Carb 2800TR, Perkin-Elmers Life Science). At the 16th minute, the atrium was stimulated at 5 Hz for 1 min (first stimulation train, S1). The solution was changed at the 27th minute to one containing a drug (see individual result for identity). A second stimulation was applied at the 49th minute for 1 min (second stimulation train, S2). At the end of the experiment, the atrium was immersed overnight in 4 U/ml papain (Sigma) and the radioactivity contained in the extract was determined (*z*). [^3^H]NE outflow was expressed as a proportional increase using the formula below:

Proportionalincrease=[DPM(y)−DPM(x)]/DPM(x)×100%

DPM(x)=DPMimmediatelybeforeelectricalstimulation

DPM(y)=DPMimmediatelyafterelectricalstimulation.

This method of calculating the [^3^H]NE was different from our previous experiment in which the presumed total tissue radioactivity (*z*) was used as denominator. However this newer method appears to be more accurate as it takes into account the reduction of radioactivity content during the course of the experiment, and obviates the variability introduced by overnight digestion and subsequent measurement of radioactivity in the tissue extract.

### 2.4 Post-synaptic beta adrenergic receptor response to exogenous norepinephrine

Double atria were isolated and transferred to a water-jacketed organ bath containing 100 ml of Tyrode solution continuously bubbled with carbogen and maintained at 37 ± 0.2 °C. The left atrium was attached to a hook at the bottom and the right atrium was attached to an isometric force transducer (Harvard Apparatus, Model 60-2997) above via 5.0 Mersilk and stretched to ∼ 0.5 g. The heart rate was triggered from the contraction and recorded via Biopac System MP100 connected to a Dell P4 computer run on the AcqKnowledge 3.7.3 software.

After equilibrating for 80 min (during which the Tyrode solution was changed every 30 min), heart rate responses to NE (cumulative dose, 0.1–5 μmol/L; Sigma) were assessed in a darkened room. After the first protocol, the double atria preparation was washed with fresh Tyrode solution for 3 to 4 times (each wash 2 min) so that the heart rate returned to within 5% of baseline heart rate. After a further equilibration of 10 min, the tissue was incubated with soluble guanylyl cyclase (sGC) inhibitor, 1H-(1,2,4)oxadiazolo(4,3-a)quinoxaline-1-one (ODQ) (10 μmol/L; Sigma) for 20 min before responses to NE was reassessed.

### 2.5 Plasma l-arg level

Free amino acids from plasma were derivatised using Applied Biosystems Model 420A PTC derivatiser and then separated (high performance liquid chromatography) and quantified with an on-line Applied Biosystems Model 130A PTC Amino Acid Analyzer. Data was stored and analysed with a Chameleon^®^ data analysis system.

### 2.6 Tissue cGMP and cAMP levels

Atrial cGMP level was determined using the Biotrak cGMP **[**^125^I] radioimmunoassay kit from Amersham Biosciences as previously described [9]. Atrial cAMP level was also determined using Biotrak cAMP[^125^I] by employing similar method.

### 2.7 Western blotting

Left ventricles were cut into small pieces on ice and put into a tube containing 200 μl of buffer (containing CelLytic buffer and cocktail protease inhibitor at a ratio of 40:1, Sigma) and 1 mm glass beads (Biospec). The tissues were homogenised for 90 s (Mini beadbeater-8, Biospec) and centrifuged (14,000 rpm) for 10 min at 4 °C. The supernatants were recovered and used for protein analysis and Western blotting.

15 μg of denatured protein from each sample was loaded into each well of a Bio-Rad Criterion XT 4–12% polyacrylamide gel. Molecular weight marker (Precision Plus Dual Colour, Bio-Rad), positive control and standards (where available) were also loaded. The proteins were separated by electrophoresis and transferred to a polyvinylidene fluoride membrane (Immobilon-P 0.45 μm, Millipore). The non-specific binding sites were blocked with 5% non-fat skim milk in phosphate buffered saline (Fisher) containing 0.05% Tween-20 (Sigma) (PBST). The membrane was then probed with a primary antibody (nNOS, Santa Cruz; eNOS, Transduction Laboratory; sGC, Sigma; TH, Sigma) overnight at 4 °C. On the following day, the membrane was rinsed twice and then washed 3 times with PBST (10 min each wash) before incubation with a suitable secondary antibody conjugated with horseradish peroxidase for 1 h at room temperature. The membrane was rinsed twice again and washed 3 times with PBST (10 min each wash). The relevant protein signal was amplified with luminol based chemiluminescence (Western Lightning Plus, Perkin Elmer Life Science) and detected using light-sensitive film (CL-XPosure film, Pierce). The film was digitised and the relative densities were determined with a computer software (UN-SCAN-IT, gel 6.1). The results were normalised to β-actin that served as loading control (detected with mouse anti-β-actin monoclonal antibody, Abcam).

### 2.8 nNOS activity assay

The activity of NOS in left ventricles was determined using NOS*detect*^®^ assay kit and protocol from Stratagene. This assay is based on the conversion of l-arg to l-citrulline by NOS in a stoichiometric way. l-citrulline can be separated from l-arg using an ion exchange column/resin. By using a radiolabelled l-arg (universally labelled ^14^C, Amersham Bioscience), the rate of its conversion to l-citrulline was determined by measuring the radioactivity of the separated labelled fraction of l-citrulline. By performing the assay in the absence and presence of nNOS inhibitor (N-[(4S)-4-amino-5-[(2-aminoethyl)amino]pentyl]N′-nitroguanidine, 20 μmol/L, Sigma), the activities of nNOS could be estimated. The assay was carried out in the presence of 5 μmol/L arginase inhibitor, N-hydroxy-nor-l-arginine (Calbiochem) as l-arg can be converted to l-citrulline via alternative pathway involving arginase [16].

### 2.9 Statistical analysis

Data are presented as mean ± S.E.M. For comparisons among more than 2 groups, one way analysis of variance (ANOVA) was used to compare the means with post-hoc Tukey's HSD test to assess individual significance. Paired *t* test was used to compare the results before (S1) and after application of inhibitor (S2) in the evoked NE release experiments. Statistical significance was accepted at *p* < 0.05.

## 3 Results

### 3.1 Animal characteristics

**Fig. 1:**
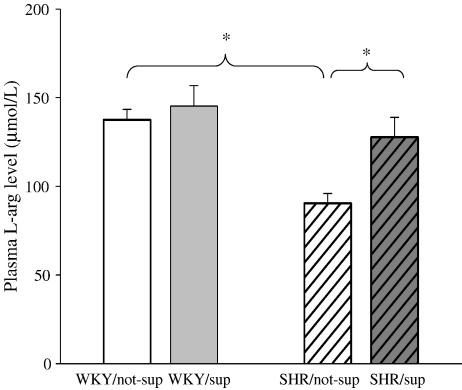
The SHR/not-sup group (*n* = 10) had a significantly lower plasma l-arg than WKY/not-sup group (*n* = 13), but it increased to a comparable level following l-arg supplementation (*n* = 10). ⁎*p* < 0.01.

SHR has higher total ventricular weight than WKY and this was not altered by l-arg feeding (Table 1). No difference in mean arterial blood pressure or heart rate between supplemented SHR (SHR/sup, 173 ± 6 mmHg, 349 ± 11 bpm; *n* = 13) and non-supplemented SHR (SHR/not-sup, 180 ± 5 mmHg, 352 ± 7 bpm; *n* = 11) was observed. Plasma l-arg level was significantly lower (*p* < 0.01) in the SHR/not-sup group (90.5 ± 5.4 μmol/L, *n* = 10) compared with the WKY/not-sup group (137.2 ± 6.1 μmol/L, *n* = 13) but it increased to a comparable level following l-arg supplementation (Fig. 1). l-arg feeding did not increase the plasma l-arg level in the WKY significantly.

### 3.2 Atrial cGMP and cAMP levels in supplemented and non-supplemented SHR and WKY

**Fig. 2:**
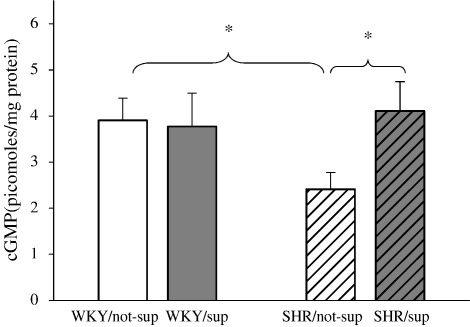
SHR has lower atrial cGMP compared with WKY but this was increased with l-arg feeding. ⁎*p* < 0.05.

We found that atrial cGMP was lower in the SHR control group compared with the WKY control group (2.4 ± 0.4 pmol/mg vs 4.4 ± 0.5 pmol/mg), but increased to 4.1 ± 0.5 pmol/mg protein (*n* = 10, *p* < 0.05) following l-arg oral supplementation (Fig. 2). We could not detect a significant difference in atrial cAMP levels among the groups (data not shown).

### 3.3 Proportional evoked NE increase in SHR and WKY

**Fig. 3:**
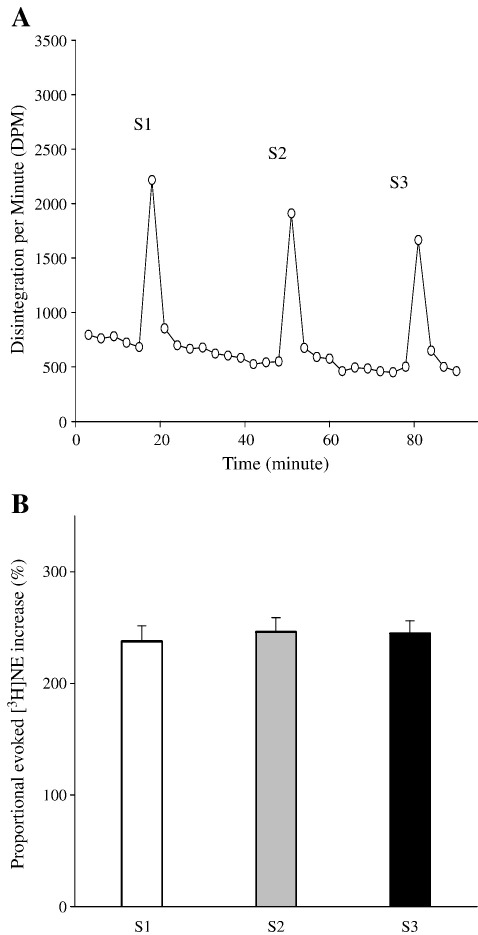
(A) Typical raw data trace from time control experiment (WKY only) showing measurement of [^3^H] NE release from isolated right atria in response to 5-Hz electrical field stimulation. S1, S2 and S3 represent the first, second and third stimulation respectively. (B) Proportional [^3^H]NE increase were similar from S1 to S3 (*n* = 4, all WKY).

**Fig. 4:**
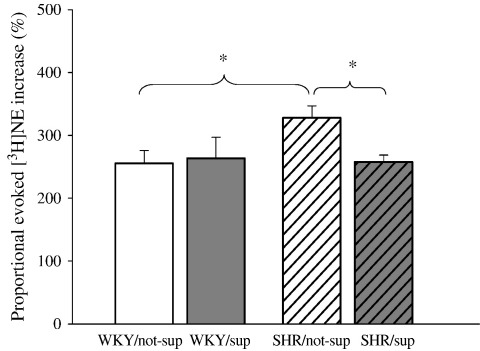
Non-supplemented SHR (*n* = 19) has higher proportional evoked [^3^H]NE increase compared with non-supplemented WKY (*n* = 14). l-arg feeding reduced the proportional evoked [^3^H]NE increase in the SHR (*n* = 24, *p* < 0.01) but did not appear to have significant effect on the WKY (*n* = 6). All refer to first stimulation, S1. ⁎*p* < 0.01.

Fig. 3 shows that proportional increase of evoked [^3^H]NE following electrical field stimulation was stable for the duration of the experiment. There was no difference among S1, S2 and S3, indicating that there was no time dependent change (Fig. 3B). Proportional evoked [^3^H]NE increase in the non-supplemented SHR (*n* = 19) was 28% higher than the non-supplemented WKY (*n* = 14, *p* < 0.01), but was reduced with l-arg feeding (*n* = 24, *p* < 0.01) (Fig. 4).

### 3.4 Soluble guanylyl cyclase inhibitor reversed the effect of l-arg feeding on NE release in the SHR

**Fig. 5:**
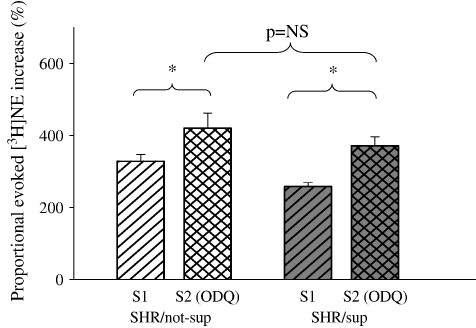
Addition of ODQ increased proportional electrically evoked [^3^H]NE release (S2) in both supplemented SHR (*n* = 6) and non-supplemented SHR (*n* = 7), but relatively more so in the l-arg supplemented group. ⁎*p* < 0.01 with paired *t* test.

Addition of a soluble guanylyl cyclase (sGC) inhibitor, 1H-(1,2,4)oxadiazolo(4,3-a)quinoxaline-1-one (ODQ, 10 μmol/L, Sigma), to the organ bath suffusate prior to second stimulation (S2) caused a greater increase of evoked [^3^H]NE release in the l-arg fed SHR (*n* = 6) compared with the non-fed SHR (*n* = 7) (*p* < 0.01 when compared with respective S1 in paired *t* test) such that the two became no longer significantly different (Fig. 5).

### 3.5 Effects of selective nNOS inhibitor on evoked NE release

Highly selective nNOS inhibitor, N-[(4S)-4-amino-5-[(2-aminoethyl)amino]pentyl]N′ nitroguanidine (5 μmol/L, Sigma), increased proportional evoked [^3^H]NE release (S2) significantly in supplemented SHR (from 264 ± 22% to 283 ± 25%, *n* = 8), supplemented (264 ± 33% to 299 ± 32%, *n* = 6) and non-supplemented WKY (276 ± 24% to 304 ± 26%, *n* = 10) (*p* < 0.05 when compared with respective S1 in paired *t* test), but not in non-supplemented SHR (from 345 ± 39% to 360 ± 42%, *n* = 8; *p* = 0.17).

### 3.6 Post-synaptic β_1_ adrenergic receptor response to exogenous norepinephrine in SHR

**Fig. 6:**
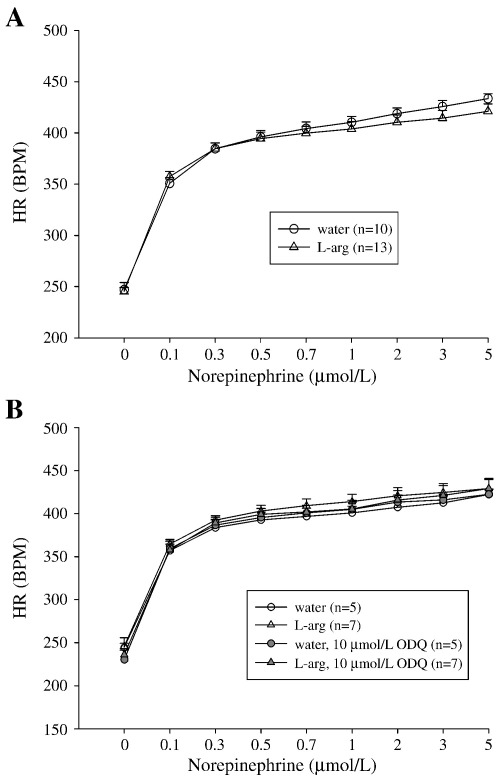
(A) In-vitro heart rate response of double atrial preparation to exogenous norepinephrine (0.1–5 μmol/L). (B) Similar result with and without incubation with ODQ.

In-vitro heart rate response of the double atrial preparations to exogenous NE (0.1–5 μmol/L) was similar between l-arg fed (*n* = 13) and non-fed SHR (*n* = 10), and was not affected by 20 min incubation with ODQ (10 μmol/L) (Fig. 6).

### 3.7 Western blotting and nitric oxide synthase activities

**Fig. 7:**
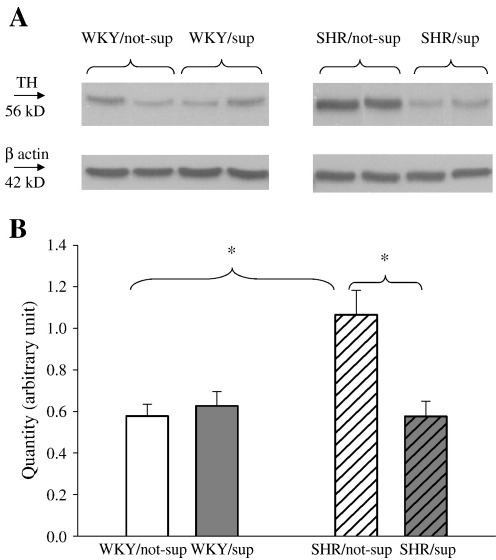
(A) Representative bands of tyrosine hydroxylase (TH) and corresponding β-actin as loading control. (B) Non-supplemented SHR has higher myocardial TH contents than WKY and supplemented SHR (⁎*p* < 0.05, *n* = 6 for each).

We found that non-supplemented SHR had a significantly higher myocardial tyrosine hydroxylase protein compared with non-supplemented WKY (*p* < 0.05, *n* = 6 in each). l-arg feeding reduced the tyrosine hydroxylase in SHR (*p* < 0.05) but not in WKY (*n* = 6 in each) (Fig. 7). We did not observe a significant difference in myocardial protein levels of eNOS, nNOS or sGC among supplemented and non-supplemented SHR and WKY (*n* = 6 in each, data not shown). The nNOS activity measured in-vitro based on l-citrulline conversion was similar between non-supplemented WKY (61 ± 29 fmol l-citrulline/mg protein/min, *n* = 4), non-supplemented SHR (75 ± 15 fmol l-citrulline/mg protein/min, *n* = 6) and supplemented SHR (72 ± 16 fmol l-citrulline/mg protein/min, *n* = 6) (*p* = NS).

## 4 Discussion

There are three novel findings from this present study. First, we demonstrated that l-arg supplementation reduced the peripheral sympathetic hyperactivity in the SHR by attenuating evoked NE release from the right atria. Secondly, this effect is mediated at least in part via an increase in the l-arg–NO–cGMP signalling (by augmenting l-arg availability), and the associated reduction in myocardial tyrosine hydroxylase protein levels. Finally, the effect of l-arg supplementation on peripheral cardiac noradrenergic neurotransmission is a pre-synaptic one, as evidenced by lack of differences between treated and untreated SHR in in-vitro heart rate responses to exogenous norepinephrine.

Most published l-arg studies have examined the effect on platelet aggregation, endothelial function and atheroma formation [12,15,17]. Few studies have assessed its impact on autonomic function, and these employed intravenous administration that may have confounding non-NO effects such as hormones secretion, pH increase and high osmolality [14,18,19] secondary to supraphysiological plasma l-arg concentration. Dietary l-arg supplementation circumvented these potential issues as the increase in plasma l-arg level is relatively lower in the physiological range where this non-NO effect has not been reported to occur. In this study, we showed that oral l-arg supplementation could decrease the peripheral cardiac sympathetic hyperactivity in the SHR to a comparable level seen in the WKY. In addition, l-arg supplementation increased atrial cGMP to levels similar to that of the WKY. l-arg is the sole substrate of the enzyme NOS, with NO as the immediate but labile product. NO in turn activates sGC to synthesise cGMP, the key secondary messenger of this signalling system [20]. There is now an abundance of evidence supporting the notion that the l-arg–NO–cGMP pathway plays an important neuromodulatory role in cardiac autonomic nervous system; augmenting the vagal arm and inhibiting the sympathetic counterpart [21,22]. More importantly in this study, we showed that bath application of a sGC inhibitor (ODQ) resulted in a higher increase in the evoked NE release in the l-arg treated SHR than non treated SHR, suggesting that this pathway had been augmented by l-arg supplementation. Similarly, the highly selective nNOS inhibitor increased the evoked NE release (S2) in the supplemented SHR but not in the non-supplemented SHR when compared with S1. Although the possibility of a non-NO effect could not be excluded completely, taken together, these results support the notion that l-arg feeding had augmented the l-arg–NO–cGMP pathway with a resultant decrease in evoked NE release. We cannot however, rule out the possibility that the reduction in evoked NE release may be an indirect result of enhanced parasympathetic neurotransmission by the augmented NOS–cGMP pathway. In addition, the conclusion arrived here is based on ex-vivo data, and therefore it remains to be established whether this is applicable to the in-vivo setting.

One of the most intriguing and significant findings in this study is the reduction of myocardial tyrosine hydroxylase level in the SHR following l-arg supplementation. To our knowledge, this is the first study to demonstrate this feature, although it is known that the SHR has higher tyrosine hydroxylase contents in key organs when compared with normotensive controls [23,24]. This observation provides a link to reduced NE synthesis following l-arg supplementation in the SHR. One possible explanation is cGMP activating phosphodiesterase-2 (PDE-2) resulting in increased hydrolysis of cAMP [25,26]. PDE-2 is known to exist in myocytes, central nervous system and stellate ganglia innervating the heart [25,27,28]. Lowering of cAMP will cause less interaction with cAMP responsive element domain of tyrosine hydroxylase gene promoter, thus reducing its expression [29]. However, we did not find that atrial cAMP levels were altered significantly by l-arg supplementation in either WKY or SHR groups. This would suggest that modulation of cAMP levels was not involved. Nonetheless, we cannot completely rule this out because localised changes of compartmentalised cAMP, a feature which has been well described [30,31], would not have been detected by measurement of total cAMP in atrial tissue.

l-arg levels should not be rate limiting to NO synthesis based on its pharmacokinetics [10,19]. Despite this, l-arg supplementation has been shown to improve endothelial function, reduce atherosclerosis progression, and augment cardiac vagal function; all consistent with enhanced NO synthesis [14,17]. We believe that this paradoxical observation, including the result of the current study, can probably be best explained by NOS compartmentalisation (limiting its access to l-arg), and a relative l-arg deficiency in diseased states from elevated endogenous NOS inhibitor such as asymmetric dimethylarginine (ADMA), rather than an absolute one [19,32,33]. We did not measure ADMA levels in this study although others have shown that plasma ADMA concentrations are higher in hypertensive subjects than normotensive ones [34,35]. Interestingly we found that plasma l-arg level was lower in the SHR when compared with the WKY, but this was increased to a comparable level following l-arg supplementation. In addition, we also found that l-arg supplementation had little effect on the WKY (either evoked NE release or cGMP levels). This was in keeping with other studies that showed that negative results were more likely to be obtained when healthy subjects were fed l-arg [12,19]. It is not entirely clear why there was no significant increase in plasma l-arg in the WKY following supplementation. Increased breakdown by hepatic arginase (whose activity is dependent on l-arg concentration) and increased renal excretion (when renal tubular reabsorption threshold is exceeded) may prevent further increase in plasma l-arg level beyond a certain set point [36,37]. It is possible that this set point is close to the normal plasma concentration of l-arg in WKY, although neither this nor other studies have looked at this pharmacokinetic question specifically in rats.

l-arg supplementation did not alter myocardial nNOS, eNOS or sGC protein levels in the SHR which may seem inconsistent with the increase in the cGMP brought about by l-arg supplementation. However, l-arg supplementation served only to provide additional substrate for NO production which in turn stimulated sGC to generate more cGMP. nNOS activity was also unchanged in the l-arg treated SHR, when measured in-vitro; consistent with the overall concept on the role of l-arg supplementation. We did not measure the amount of NO produced in-vivo because of its labile nature, but had shown that l-arg feeding increased the more stable secondary messenger cGMP in the SHR.

In the double atrial experiments, we observed that l-arg supplementation did not affect the heart rate response to exogenous NE in the SHR. In other words, the effect of l-arg supplementation on cardiac noradrenergic neurotransmission is a pre-synaptic one and did not appear to change the post-synaptic beta adrenergic receptor driven excitability. Gene transfer of nNOS with an adenovirus to the heart has been found to attenuate evoked NE release, a pre-synaptic feature, via upregulation of NOS–sGC pathway [9]. It also reduces the heart rate response of SHR atria to exogenous NE, possibly via NO–cGMP reducing L-type calcium current in the sinoatrial nodal cells [38]]. The result of the latter was probably related to the fact that nNOS gene transfer with a viral vector is a more powerful tool in augmenting NOS–cGMP pathway, although other non-NO factor could not be excluded.

Basal heart rate or arterial blood pressure did not differ between supplemented and non-supplemented SHR when measured under anaesthesia. From published literatures [39–41], it is difficult to predict whether the result would be different if the measurements were taken in conscious telemetered rats, given the duration of the supplementation was for one week only. However, the absence of difference was consistent with the basal heart rate findings of the isolated double atrial preparations. Taken together (including the evoked NE results), this implied that l-arg, through the NO–cGMP pathway, probably plays a modulatory role during sympathetic nerve stimulation rather than under basal resting condition.

### 4.1 Clinical perspective

l-arg has been used widely as a potential therapeutic tool in both human and animal studies. Two recent clinical trials on patients with myocardial infarction have yielded conflicting results [42,43]. The study [43] which was stopped prematurely because of safety concerns failed to demonstrate any increase in plasma l-arg following supplementation and did not measure markers of NO production. This raised the question whether any effects observed could be confidently attributed to the l-arg treatment. Our study of l-arg supplementation on the SHR is significantly different, because it demonstrates both an increase in plasma l-arg as well as augmentation of nNOS–sGC–cGMP pathway with a reduction in evoked NE release. Moreover the SHR is from a homogenous population; the same is unlikely for the human subjects. Whether this intervention could restore autonomic balance and thus improve outcome in patients with hypertension will require a randomised clinical trial in a well controlled well selected clinical population. Accumulating evidence suggest that certain groups such as those with elevated endogenous NOS inhibitor ADMA and impaired l-arg–NO metabolism may be more likely to benefit from l-arg supplementation [44]. Therefore inclusion of such criteria in patient selection for any future clinical studies may help to ensure a high probability of success.

## Figures and Tables

**Table 1:** Animal characteristics.

	SHR/not-sup *n* = 19	SHR/sup *n* = 24	WKY/not-sup *n* = 14	WKY/sup *n* = 6
Body weight (g)	336 ± 7	332 ± 8	320 ± 6	311 ± 4
Ventricular weight (g)	1.10 ± 0.03⁎	1.10 ± 0.03⁎⁎	0.95 ± 0.02⁎	0.91 ± 0.01⁎⁎
Ventricular/body weight × 100	0.327 ± 0.004⁎	0.331 ± 0.004⁎⁎	0.300 ± 0.003⁎	0.290 ± 0.001⁎⁎
Supplemented l-arg intake (g l-arg/kg body weight/day)	–	0.92 ± 0.03	–	0.85 ± 0.03

⁎ *p* < 0.01 for comparison between SHR and WKY (both not-supplemented).

⁎⁎ *p* < 0.01 for comparison between SHR and WKY (both supplemented).

## Glossary

ADMA: asymmetric dimethylarginine
cGMP: cyclic guonosine monophosphate
DPM: disintegration per minute
l-arg: l-arginine
NE: norepinephrine
NO: nitric oxide
NOS: nitric oxide synthase
eNOS: endothelial nitric oxide synthase
nNOS: neuronal nitric oxide synthase
ODQ: 1H-(1,2,4)oxadiazolo(4,3-a)quinoxaline-1-one
PDE-2: phosphodiesterase-2
sGC: soluble guanylyl cyclase
SHR: spontaneously hypertensive rats
TH: tyrosine hydroxylase
WKY: Wistar Kyoto rats

## Footnotes

1 See Glossary.
